# The Synergistic Effect of Ionic Liquid-Modified Expandable Graphite and Intumescent Flame-Retardant on Flame-Retardant Rigid Polyurethane Foams

**DOI:** 10.3390/ma13143095

**Published:** 2020-07-10

**Authors:** Yongjun Chen, Yuanfang Luo, Xiaohui Guo, Lijuan Chen, Demin Jia

**Affiliations:** 1 College of Materials Science and Engineering, Key Lab of Guangdong Province for High Property and Functional Macromolecular Materials, South China University of Technology, Guangzhou 510641, China; psyfluo@scut.edu.cn (Y.L.); msgxh@mail.scut.edu.cn (X.G.); psdmjia@scut.edu.cn (D.J.); 2 Center for Advanced Analytical Science, c/o School of Chemistry and Chemical Engineering, Guangzhou University, Guangzhou 510006, China

**Keywords:** polyurethane foam, expandable graphite, ionic liquid, flame-retardant, synergistic flame-retardant

## Abstract

In this study, a nitrogen–phosphorus intumescent flame-retardant 3-(N-diphenyl phosphate) amino propyl triethoxy silane (DPES), the ionic liquid (IL) of 1-butyl-3-methyl-imidazole phosphate, and a phosphorous-containing ionic liquid-modified expandable graphite (IL-EG), were synthesized, and their molecular structures were characterized. The flame-retardant rigid polyurethane foams (RPUFs) were compounded with synergistic flame-retardant IL-EG/DPES to study the effects of the combination IL-EG and DPES on the pore structure, mechanical properties, thermal decomposition behavior and thermal decomposition mechanism of RPUF. The results showed that IL-EG/DPES had good thermal stability, and an excellent expansibility and char yield. The flame-retardant RPUF, modified with IL-EG and DPES at the ratio of 1:1, had a relatively uniform pore size, the highest compressive strength, and an excellent flame-retardant performance due to the form interwoven hydrogen bonds between IL-EG and DPES, as well as the new synergistic flame-retardant coating on the RPUF surface to restrict the transfer of gas or heat into the PU matrix.

## 1. Introduction

Rigid polyurethane foams (RPUFs) have been widely applied in thermal insulation, sound insulation and shock resistance, etc., on account of their foam structure, low thermal conductivity, excellent specific strength and low density. However, duo to the properties of inflammability and the rapid spread of flames on RPUFs, the addition of flame-retardants to RPUFs is essential, especially halogen-free flame-retardants. In addition, RPUFs flame-retardants are generally enhanced by additives and reactive flame-retardants. Various flame-retardant additives are based on phosphorous, nitrogen and inorganic compounds, such as modified red phosphorus [1], expanded graphite (EG) [2,3], ammonium polyphosphate [4,5], melamine [3] and intumescent flame-retardants [6,7]. Recently, silicone flame-retardants have acquired increasing applicability for their high efficiency, environmental friendliness, antidripping effects and smoke suppression. Silicone can improve the flame-retardancy of polymers along with other properties, such as processing and thermal stability [8,9,10]. In silicone flame-retardants, silicone migrates to the polymer surface during combustion to form a protective char layer [via the formation of silicon–oxygen (Si-O) and silicon–carbon (Si-C) bonds] that can inhibit combustion [4,11]. Silicone flame-retardants are rarely used alone, and are typically used in combination with other flame-retardants. Two types of combination flame-retardants are most commonly used [12,13,14,15]. The first is the combination of a silicone flame-retardant with other flame-retardants to produce a synergistic flame-retardant [5,7,16]. In the second type, molecular design is used to introduce phosphorus (P), nitrogen (N), boron (B) and other flame-retardant elements into silicone flame-retardant molecules, to achieve a synergy among the various flame-retardant elements [17,18]. Li et al. used hydroxyl-terminated polysiloxane, N-(*β*-aminoethyl)*-γ*-amino-propylmethyldimethoxy silane and phosphorus oxychloride as raw materials to synthesize a flame-retardant containing Si, N and P. For a Si:N:P mass ratio of 2.0:1.0:4.4, the limiting oxygen index (LOI) of the polymer with the lowest FR content increased from 24.0% to 26.5%, which was compared with the polymer containing only one or two flame-retardant elements and a higher FR content [19]. The significant increase in the LOI indicated good synergy among the three flame-retardant elements, while maintaining the mechanical properties of the polymer matrix [20,21,22,23].

Silane coupling agents are a group of organosilicon compounds that can simultaneously interact with polymer materials and inorganic fillers [9,24]. These compounds contain two functional groups in the molecule chains, which can form a molecular bridge between the polymer matrix and the inorganic filler through chemical reaction or physical interaction, in order to improve the rubber–filler interfacial interaction as well as the mechanical properties [25,26,27].

As a new type of functional carbon material, expandable graphite (EG) is a loose and porous worm-like substance, obtained from natural graphite after intercalation, washing, drying and high-temperature expansion [28,29]. Expandable graphite can expand 150~300 times in volume in high temperatures, changing from flaky to worm-like, and obtaining a loose, porous, curved and expanded surface structure. Furthermore, the surface energy of EG is increased, and the force of the adsorbing flake graphite is enhanced. EG has not only the excellent properties of natural graphite such as heat- and cold-resistance, corrosion resistance and self-lubrication, but also has the properties of softness, compression resilience, absorbency, ecological environment coordination, biocompatibility and electromagnetic shielding, which natural graphite does not have. Li et al. prepared a flame-retardant RPUF, with incorporated phosphorus and nitrogen containing flame-retardant 1,4-bis(Diethyl methylenephosphonate) piperazine (BDEMPP) and EG. The fire-retardant properties of RPUFs were improved due to the barrier effect of EG and residual char [3]. Furthermore, the larger particle size of EG exhibited a superior synergistic effect and larger expansion ratio, as it formed a compacted, united protective layer, which enhanced the flame-retardant property of RPUFs [2].

In this study, we have synthesized a novel halogen-free intumescent flame-retardant, namely, 3-(N-diphenyl phosphorous) amino-propyl triethoxysilane (DPES). Besides this, DPES, combined with ionic liquid-modified expandable graphite (IL-EG), has been applied to RPUF. The effects of IL-EG and DPES on the mechanical properties of RPUF were studied. Thermogravimetric analysis (TGA) was used to study the thermal degradation of RPUF modified by IL-EG/DPES. The influence of the dosage of compound flame-retardants on the RPUF flame retardancy was studied by performing LOI measurements and cone calorimetry (CONE), and the char residue was analyzed by scanning electron microscopy (SEM).

## 2. Materials and Methods

### 2.1. Materials

Industrial-grade polyether polyol (brand HF-4110H), with a hydroxyl value of 430 ± 30 mg KOH/g and a viscosity of 1500–2000 mPa/s (25 °C), and industrial-grade polyether polyol (brand HF-4110), with a hydroxyl value of 430 ± 30 mg KOH/g and a viscosity of 4500–6500 mPa/s (25 °C), were purchased from Hengfeng Polyurethane Industry Co., Ltd. (Zhejiang, China). Expandable graphite (EG) (brand ADT249), with the initial expansion temperature 165 °C, was purchased from Shijiazhuang Kepeng Flame-retardant Material Factory (Shijiazhuang, China). Polyisocyanate (PAPI) with an NCO content of 31%, a viscosity of 200 mPa/s and a functionality of 2.7 was purchased from NCM Hersbit Chemical Co., Ltd. (Shanghai, China) Industrial-grade 3-amino-propyl triethoxysilane (APTES) was purchased from Guangzhou Kangoushuang Trading Co., Ltd.(Guangzhou, China) Industrial-grade diphenylphosphinous chloride (DPCP) was purchased from Jinjinle Chemical Co., Ltd.(Shanghai, China) Analytical-grade triethylamine (TEA) was purchased from Guangzhou Chemical Reagent Factory. An industrial-grade 33% solution of tri-ethylenediamine in dipropylene glycol (A33) was purchased from Shanghai Hongpu Chemical Co., Ltd (Shanghai, China). Industrial-grade silicone oil (AK-8805) was purchased from Guangzhou Liangkun Chemical Co. (Guangzhou, China) Glycerin (GI) and toluene, both analytical grade, were purchased from Guangzhou Chemical Reagent Factory (Guangzhou, China). Deionized (DI) water and IL-EG were prepared in the laboratory.

### 2.2. Preparation of DPES

**Figure 1:**
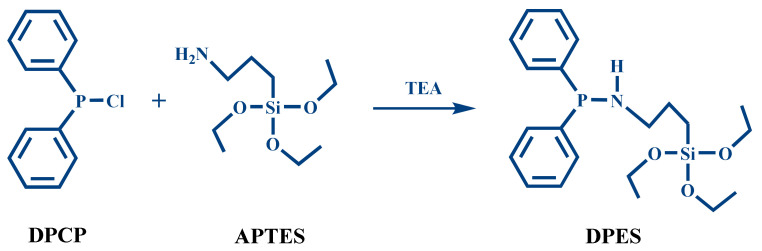
The synthesis route of DPES.

The synthetic procedure of intumescent flame-retardants (IFR) DPES was as follows. First, 5.02 g of APTES and 100 mL toluene were added to a 250-mL four-neck round-bottom flask with nitrogen, and then 0.2 g TEA was added under continuous stirring. A solution of 5.00 g of DPCP in 50 mL toluene was then slowly added to the flask through a constant-pressure dropping funnel for 2 h. When the addition was complete, the temperature was raised to 85 °C, and the reaction continued under nitrogen for 10 h. After the reaction was completed, the mixture was filtered, washed with cyclohexane for three times, and then was vacuum distilled. The novel flame-retardant synergist DPES, with a yield of approximately 91%, was obtained. The synthesis route is shown in Figure 1.

### 2.3. Preparation of IL-EG

First step: N-methylimidazole (8 mL, 0.1 mol) was added to a three-neck flask and heated to 70 °C with several drops of 1-bromobutane (13 mL, 0.12 mol) with a mechanical stirrer and a reflux condenser. The mixture was reacted under 70 °C for 20 h. After the reaction was completed, the mixture was filtered, washed with ethyl acetate three times, and then was vacuum distilled to get the purified intermediate product named [Bmin]-Br. Second step: 13 g of the prepared intermediate product [Bmin]-Br was added in a wild-mouth bottle and we slowly added 20 mL KOH solution. The mixture was reacted under room temperature for 10 h. After the reaction was completed, the mixture was filtered, washed with methyl alcohol several times and then was vacuum distilled to get the hydroxide-1-butyl-3-methyl imidazole. Finally, a certain ratio of H_3_PO_4_ was added to the hydroxide-1-butyl-3-methyl imidazole to adjust to the weak acidity. After removing the solvent by reduced pressure distillation, the 1-butyl-3-methyl-imidazole phosphate (marked IL) was obtained. The characterization of the structures of [Bmin]-Br and IL are shown in Figure S1.

**Figure 2:**
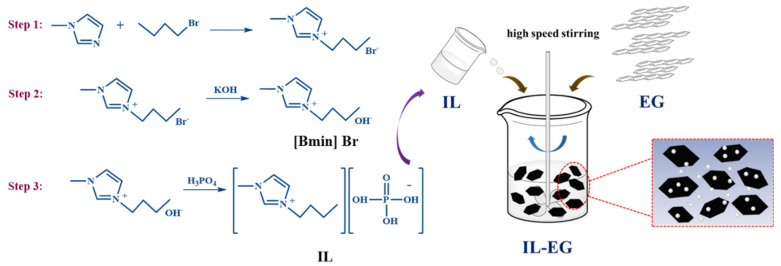
The synthesis route of IL and the illustration of the fabrication of the IL-EG.

The expandable graphite (EG) and IL prepared in the laboratory were physically mixed in a 1:1 ratio under high-speed stirring. In order to ensure the uniformity of the mixing, mixing 3 times, 30 s each time, is necessary. Then, the mixture of EG and IL is placed in a beaker and post-processed in a microwave oven to obtain ionic liquid modified expandable graphite (IL-EG). The synthesis route of IL and the illustration of the fabrication of the IL-EG are shown in Figure 2.

### 2.4. Preparation of Flame-Retardant RPUF

The flame-retardant RPUFs were prepared using a one-step method. Specified quantities of HF-4110H, HF-4110, A33, AK-8803, H_2_O and the IL-EG and DPES flame-retardants were added to a 1000-mL plastic beaker and mixed evenly under the high-speed disperser. PAPI was rapidly added to the mixture, and stirring was continued for 5 min. When the bubbles escaped from the mixture and the color of mixture turned white, the mixture was rapidly injected into a mold (300 mm × 300 mm × 100 mm) for foaming, expansion and molding. After forming for 10 min, the foam was demolded, and the prepared foam was cured at 80 °C for 5 h and then cut into appropriate shapes and sizes for tests. The formulas of RPUF/IL-EG composites were listed in Table 1.

### 2.5. Characterization

The FTIR spectrum was recorded on a Nicolet Magna 360 spectrometer by KBr disk method, scanned from 4000 cm^−1^ to 400 cm^−1^ with a resolution of 4 cm^−1^. Proton nuclear magnetic resonance (^1^H NMR) was performed on a Bruker AVANCE III 600 MHz spectrometer (Bruker Corporation, Billerica, Massachusetts, USA). The content of each element in DPES was detected by an ELEMENTAR elemental analyzer (Elementar Analysensysteme GmbH, Langenselbold, Germany). The foam structures of the RPUF/IL-EG composites were observed by a Carl Zeiss LEO 1530VP scanning electron microscopy (SEM) (LEO Company, Oberkochen, Germany) and a BX51 Polarizing optical microscope (Olympus Company, Tokyo, Japan), respectively. The macro morphology of the residues from the cone calorimeter was observed with an EOS400D digital camera (Canon, Tokyo, Japan). Thermogravimetric analysis (TGA) was conducted under nitrogen atmosphere with a TA Q5000 (TA Instruments, Shanghai, China) at a heating rate of 10 °C/min from 30 to 700 °C. To trace the typical quenching fragments of the composite, the thermogravimetry coupled to infrared spectrum analysis was employed with a Perkin-Elmer STA6000 simultaneous thermal analyzer (PerkinElmer, Waltham, MA, USA) and a Perkin-Elmer FRONTIER fourier transform infrared spectrometer (PerkinElmer, Waltham, MA, USA). Nitrogen was utilized as the carrier gas for the volatile products at flow rate of 50 mL/min, and temperature was scanned from 40 °C to 800 °C at a heating rate of 20 °C/min.

Cone colorimeter tests were done using a cone calorimeter (Fire Testing Company, East Grinstead, West Sussex, UK), at a heat flux of 35 kW/m^2^ with specimen dimensions of 100 × 100 × 25 mm^3^ according to ISO 5660 standard [28]. Horizontal combustion tests were done using a CZF-3 horizontal and vertical combustion tester (Nanjing Jiangning Analytical Instrument Co., LTD. Nanjing, China) with specimen dimensions of 125 × 13 × 10 (L × W × H) mm^3^ according to ISO1210: 1992 standard [30]. The Roell Z010 universal material testing machine from the Zwick company (Ennepeta, Germany) was used to test and analyze the compression performance of PU foams according to ASTM D1621-2016 standard [28], and the sample dimensions were 50 mm × 50 mm × 50 mm.

## 3. Results and Discussion

### 3.1. Structural Characterization of DPES

**Figure 3:**
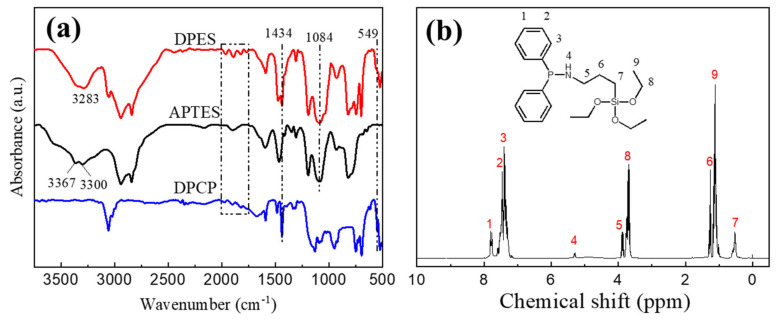
(**a**) FTIR spectra of DPCP, APTES and DPES; (**b**) ^1^H NMR spectrum of DPES.

Figure 3a shows the FTIR spectra of APTES, DPCP and DPES. The peaks at 1769~1961 cm^−1^ and 1434 cm^−1^ are the characteristic absorption peaks for the phenyl group, and the peak at 549 cm^−1^ corresponds to the stretching vibration of P-Cl in DPCP. Moreover, the peaks at 3367 cm^−1^ and 3300 cm^−1^ correspond to the stretching vibrations of -NH_2_ in APTES, and the peak at 1084 cm^−1^ corresponds to the Si-O stretching vibration in APTES. In DPES, the characteristic absorption peaks of phenyl and Si-O are still present, and the characteristic absorption peak of -NH_2_ is changed from the double-peak in APTES to a single peak at 3283 cm^−1^, due to the primary amine in APTES being converted into a secondary amine in the reaction. In addition, the characteristic absorption peak of P-Cl disappeared, indicating the successful reaction of DPCP with APTES. Figure 3b shows the ^1^H NMR spectrum of DPES. The chemical shifts corresponding to the hydrogen atoms in DPES are 7.79 (1H), 7.45 (2H), 7.38 (3H), 5.29 (4H), 3.82 (5H), 1.26 (6H), 0.53 (7H), 3.67 (8H) and 1.13 (9H), respectively. Both the FTIR and elemental analysis results show the successful synthesis of DPES.

Table 2 shows data from the elemental analysis of IFR DPES, including the theoretical and measured contents of C, H, N, P and Si. As shown in Table 2, the measured elemental contents are very close to the theoretical values. The structure of the synthesized DPES was thus further confirmed.

### 3.2. Effects on Pore Morphology of RPUF with Different Flame-Retardant Systems

**Figure 4:**
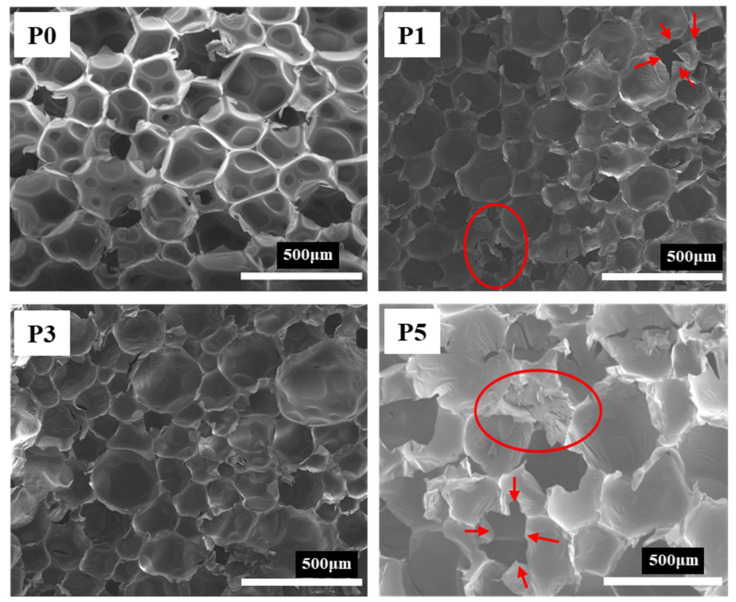
SEM morphology of RPUF with different flame-retardants.

The scanning electron microscopy (SEM) images of RPUF with different flame-retardant systems are shown in Figure 4. It can be seen that, compared with RPUF (P0), after adding flame-retardants, the PU foaming exhibits a bigger size of bubble holes and obvious edge lines. After cooling, the shrinkage of the bubble holes leads to the generation of edge wrinkles. On the other hand, merely adding the DPES sample (P1) and the IL-EG (P5) sample both reduced the integrity of the PU pores, and the phenomenon of bubble breaking was more evident than it was in RPUF, as the red arrows point out in the figure. However, with IL-EG/DPES at the ratio of 1:1 (P3), the phenomenon of bubble breaking was alleviated to a certain extent. With the increase of the IL-EG content in RPUF, the large bubble pores gradually increased, and the size uniformity decreased. This suggests that the combination of adequate IL-EG and DPES can improve the density and strength of RPUF foam.

### 3.3. Mechanical Properties of IL-EG/DPES/RPUF Composites

**Figure 5:**
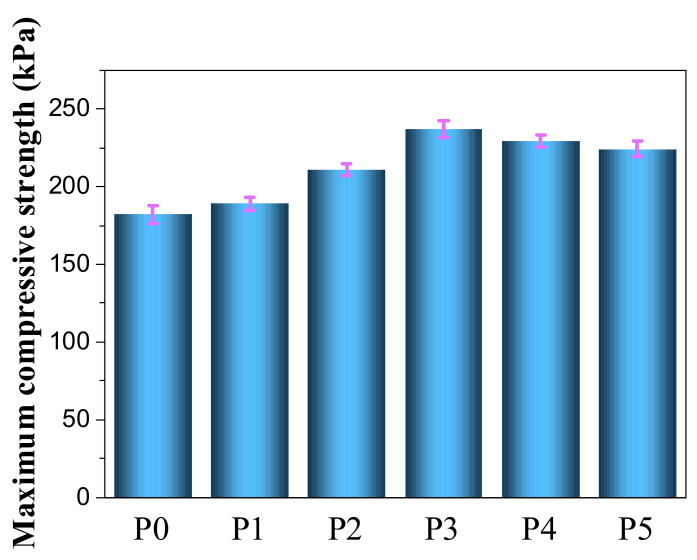
Maximum compressive strength of RPUF and its flame-retardant RPUF composites.

Figure 5 shows the compressive strengths of RPUF and the flame-retardant RPUF. For fixed total amounts of the flame-retardants, the compressive strength of RPUF was increased to varying degrees by the addition of IL-EG and the intumescent flame-retardant DPES. When adding 10 phpp each of IL-EG and DPES, the maximum compressive strength of P3 was 237 kPa, which was 30.2% higher than that of the pure RPUF. An appropriate amount of DPES improves the interfacial compatibility of IL-EG and RPUF, resulting in the uniform dispersion of IL-EG in the RPUF matrix. In addition, adding DPES enables IL-EG to bind more tightly to the RPUF matrix, resulting in the high compressive strength of the flame-retardant RPUF. The densities of each sample, from P0 to P5, were 36 kg/m^3^, 40 kg/m^3^, 43 kg/m^3^, 43 kg/m^3^, 45 kg/m^3^ and 42 kg/m^3^, respectively.

### 3.4. Thermal Degradation Behavior of Flame-Retardant RPUF

**Figure 6:**
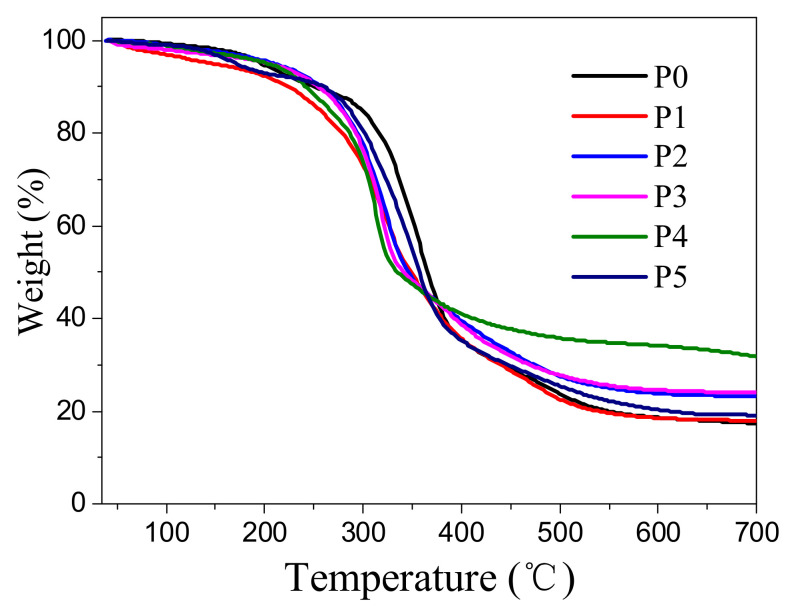
Thermogravimetric curve of different contents of IL-EG/DPES-modified flame-retardant RPUFs.

The weight loss curves of RPUF modified with different contents of IL-EG/DPES are shown in Figure 6, and the differential thermal gravity (DTG) curves are shown in Figure S2. The TG and DTG data for the various samples are shown in Table 3. The degradations of all of the RPUF composites were characterized by three stages: the first stage is below 200 °C, the second stage is between roughly 200 and 400 °C, and the third stage is between roughly 400 and 700 °C [31]. The thermal stability curve for RPUF was only modified with DPES, as P1 showed a decrease in the thermal degradation stability relative to unmodified RPUF. However, the combination of IL-EG and DPES can improve the temperature of 5% weight loss rate of RPUF to a certain extent. Increasing the IL-EG content in the system also increased the amount of char residue obtained at 700 °C [32,33]. In general, the thermal stability of the IL-EG/DPES-modified RPUF was improved over that of RPUF with the relevant IL-EG/DPES ratios, indicating the excellent synergistic effect of the two flame-retardants IL-EG and DPES. Moreover, with the increasing content of IL-EG in RPUFs, the char residues of P1 to P5 should increase gradually, in theory. However, the results of the char residue for each sample are irregular, and sample P4 shows the maximum amount of char residue. It is probably due to the synergistic effect between IL-EG and DPES. In addition, the siloxane structure of DPES can increase the residual weight after 700 °C heating.

**Figure 7:**
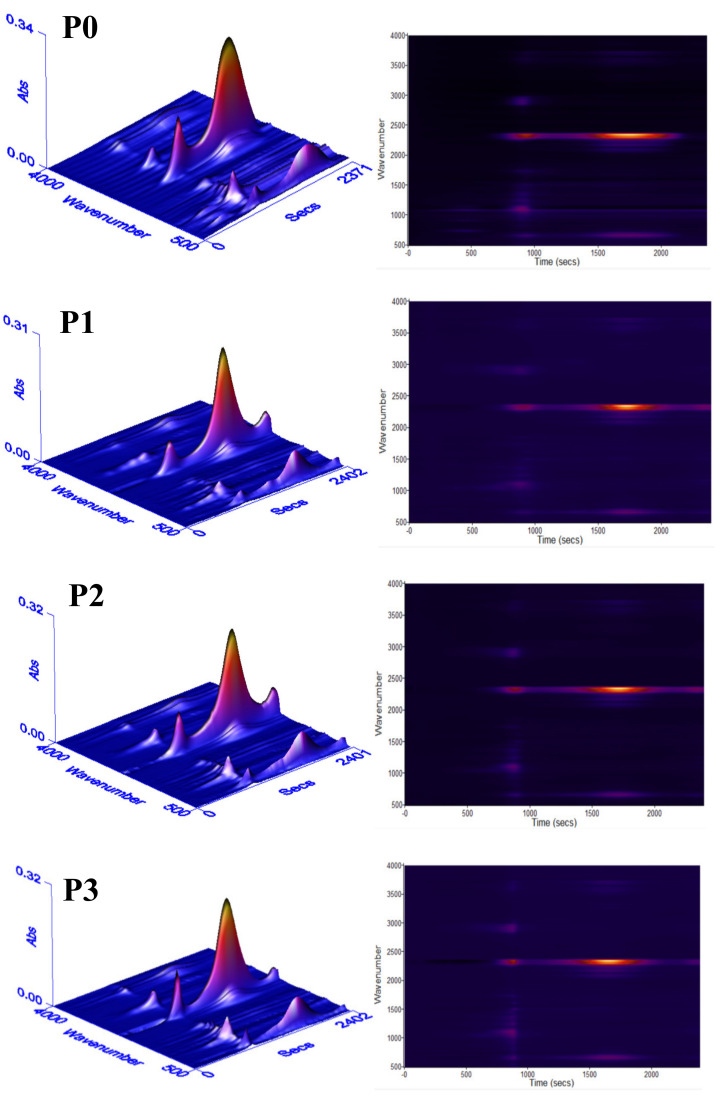

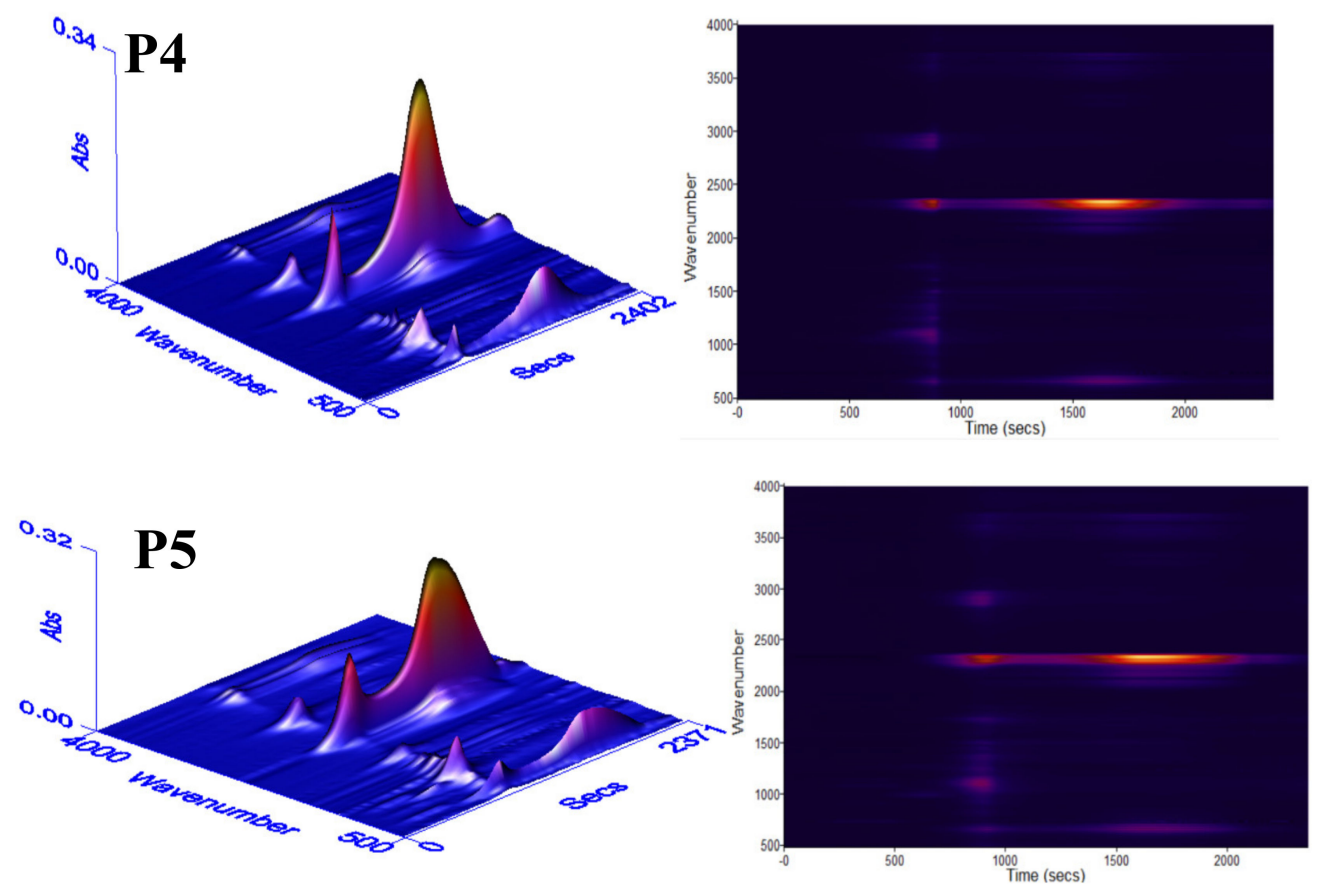
3D FTIR spectra of escaped gases from the degradation of RPUF and RPUF/IL-EG/DPES.

The decomposition process of RPUF was studied via TG-FTIR. The FTIR of gases escaping during the thermal decomposition of flame-retardant RPUFs, modified with IL-EG/DPES at different ratios, is shown in Figure 7, and the FTIR analysis of gas-escape for RPUF and IL-EG/DPES flame-retardant RPUF at the point of maximum weight loss rate is shown in Figure S1. As can be seen, compared with RPUF, the infrared absorption peak strengths of all the decomposing flame-retardant RPUFs decrease to different degrees, indicating that the volume of escaped gas decreases. By comparing with P1 and P0, it can be found that when 20 phpp DPES were added to the matrix, the decomposition of RPUF at around 200 °C was significantly changed, mainly manifesting in the fact that the ether bond absorption peak at 1120 cm^−1^, and the H-C-N absorption peak at 722 cm^−1^, were significantly weakened or even disappeared, indicating that DPES effectively inhibited the front-end thermal decomposition behavior of the RPUF. When the temperature continued to rise to 350 °C, the stretching vibration peak of the saturated C-H bond, near 2930 cm^−1^ in P1, significantly broadened, and the peak strength weakened. Meanwhile, the peak of the ester group at 1257 cm^−1^, that of the C-N group around 1220 cm^−1^~1320 cm^−1^, and the −C=O group at 1744 cm^−1^, also trended to different degrees of attenuation, indicating that DPES had an outstanding inhibitory effect on the thermal decomposition of RPUF [34]. This is due to the fact that DPES is a compounded flame-retardant of the silicone-phosphorus series. Under the effect of high temperature, organic phosphorus is first decomposed by heat to generate a viscous liquid of phosphoric acid and polyphosphoric acid. The viscous liquid covers the polymer’s surface to form a barrier layer, catalyzing the formation of carbon layer and inhibiting the influence of external heat and gas on the PU substrate. Meanwhile, the organic silicon will also decompose to form Si-C or silica, and these will migrate to the surface of the PU to accelerate the formation of the residual carbon layer, which is conducive to the development of the barrier effect. Therefore, the synergistic effect of the silicone-phosphorus series can have an excellent flame-retardant effect. In addition, phosphorus itself can also generate traps for burning the free radicals H· or OH· during the thermal decomposition process, such as PO· or HPO·, which therefore act as gas-phase flame-retardants. When the thermal decomposition temperature exceeds 550 °C, a large amount of CO_2_ and CO are generated in the system, and with the carbonization and fused ring formation of the polymer, a small molecular structure of some aromatic hydrocarbons is formed. Beyond 700 °C, DPES-modified RPUF still has incompletely carbonized polymers, which decompose and release small-molecule gasses.

### 3.5. Effect of IL-EG/DPES on RPUF Flame-Retardancy

#### 3.5.1. CONE Calorimeter Analysis

The cone calorimetric data for RPUF and the synergistic flame-retardant RPUFs with various ratios of IL-EG and IFR DPES were shown in Table 4. With the IL-EG/DPES ratio changed, the time to ignition (TTI) first increased and then decreased, whereas an initial decrease followed by an increase was observed for the peak heat release rate (PHRR), the total heat release (THR) and the total smoke release (TSR). Optimal values were obtained for all of the flame-retardance properties at an IL-EG/DPES ratio of 1:1, indicating that the combination of the two flame-retardants effectively improved the flame-retardancy of RPUF. Before the RPUF ignites completely, IL-EG can decompose and expand, whereby a large quantity of heat is absorbed, and an intumescent char layer begins to form. With the increase in the IL-EG content, the intumescent char layer becomes thicker and denser, thereby blocking the diffusion of oxygen into the interior of the RPUF matrix. DPES, as an organic IFR containing N and P elements, can thermally decompose and produce small molecules of gas, which can dilute the oxygen concentration at RPUF’s surface. When combined with IL-EG, DPES can further promote the expansion of the char layer, and increase the diffusion path length of oxygen.

**Figure 8:**
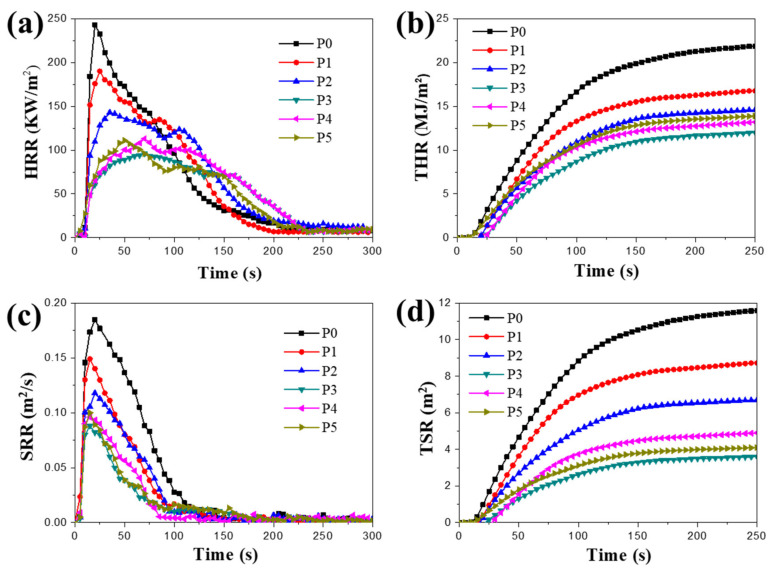
The curves of the heat release rate (**a**), the total heat release (**b**), smoke release rate (**c**) and the total smoke release (**d**) of the flame-retardant RPUF modified by IL EG/DPES.

The curves of the heat release rate (HRR), the total heat release (THR), smoke release rate (SRR) and the total smoke release (TSR) of the flame-retardant RPUF modified by IL-EG/DPES was shown in Figure 8. For settled amounts of flame-retardants, the PHRR curves (as shown in Figure 8a) first decreased and then increased with the variation ratio of the flame-retardant. The lowest PHRR was found for a 1:1 flame-retardant ratio, and it was 60.5% lower than that of RPUF. The time for the maximum heat release for the combination flame-retardant was delayed relative to that of RPUF, which effectively reduced the heat generated in combustion and improved the flame retardancy of RPUF. However, the thermal stability of the silane coupling agent is poor, and the large DPES content is not conducive to improving the flame-retardancy. In summary, the flame-retardancy of RPUF was optimized with a 1:1 ratio of IL-EG and DPES. As shown in Figure 8b, the curves of THR for the flame-retardant RPUF illustrate that when the total amount of the flame-retardant is constant, the total heat release of the flame-retardant RPUF composite first decreases, and then increases with the change of the complex ratio of IL-EG and DPES. Compared with RPUF, the THRs of the flame-retardant RPUF composites, such as P1, P2, P3, P4 and P5, decreased to 16.8 MJ/m^2^, 14.6 MJ/m^2^, 12.0 MJ/m^2^, 13.2 MJ/m^2^ and 13.9 MJ/m^2^, and were down 22.9%, 33.0%, 45.0%, 39.4% and 36.2%, respectively. This is similar to the results for the heat release rate, in which P3 had the most obvious decrease in heat release rate. The smoke generation rates (SRR) of RPUFs with different flame-retardant systems are shown in Figure 8c. It can be seen from the curves that the smoke generation rate of various composite materials reaches the maximum within 25 s of the beginning of combustion. With the extension of burning time, the SRR value of each composites increases first, and then decreases. Compared with RPUF, the peak of the SRR for flame-retardant RPUFs first decreases, and then tends to be stable. Compared with the peak smoke release rate (PSRR) of RPUF, the PSRRs of P1, P2, P3, P4 and P5 were 19.5%, 39.5%, 48.1%, 52.4% and 45.9%, respectively. This indicates that the addition of compound flame-retardants has a significant smoke suppression effect on RPUF. Figure 8d shows the TSR curves of RPUF and the flame-retardant RPUFs during combustion. Compared with RPUF (P0), the TSR of flame-retardant RPUFs decreased significantly. In addition, the TSR of compounds of IL-EG and DPES flame-retardant RPUFs decreased more significantly than that of single flame-retardant IL-EGs or DPES RPUFs. The value of TSR of RPUF at 250 s was 11.6 m^2^; after adding the compound flame-retardant, the values of the TSR of P1, P2, P3, P4 and P5 were reduced to 8.7 m^2^, 6.7 m^2^, 3.6 m^2^, 4.9 m^2^ and 4.1 m^2^, respectively, and the decrease was 25.0%, 42.2%, 69.0%, 57.8% and 64.7%, respectively. This indicates that the compound flame-retardant has a significant smoke-suppression effect on RPUF. This is due to the dense and thick carbon layer formed by the thermal expansion of IL-EG, which can effectively block the penetration of smoke and extend the propagation path to reduce the peak value of the smoke release rate and the total smoke release [28]. While DPES is in the process of pyrolysis in order to form a dense carbon layer, the formed molten material will coat the surface of the unburned substrate of the RPUF, weaken the flame momentum, and thereby reduce the combustion of the inner foam, and reduce the volatilization of fine carbon particles. This therefore increases the content of the condensed carbon layer of the RPUF substrate, and increasing the carbon rate of the layer will eventually manifest a reduction in the total amount of smoke.

#### 3.5.2. Horizontal Burning Performance

The horizontal burning data for the flame-retardant RPUFs with various IL-EG/DPES ratios are shown in Table 5. The combination flame-retardant caused the horizontal burning rate of the RPUF to initially decrease and then increase. For a 1:1 ratio of IL-EG and DPES, the horizontal burning rate of the RPUF reached a minimum value of 25.6 mm/min^−1^. In the absence of flame-retardants, the RPUF burned completely with a significant dripping effect. After being modified with the flame-retardants, the RPUF possessed excellent self-extinguishing properties, a dripping effect was unobserved, and a char layer formed on the RPUF’s surface during burning. A comparative analysis of the results showed the effectively improved flame-retardancy of the RPUF modified with the combination flame-retardant, over RPUF. This significant difference can be attributed to the action of an appropriate amount of IL in the combined system, which improves the dispersion and interfacial bonding of EG in the RPUF matrix, thereby facilitating the formation of a stable and dense expanded char layer. The simultaneous addition of DPES to the RPUF catalyzes the formation of a residual char layer, and improves the atmosphere in the combustion process, thereby slowing the burning rate until extinction. However, further increasing the IL-EG content increases the horizontal burning rate, and can reduce the DPES content, thereby decreasing the synergistic flame-retardant effect. The uneven dispersion of excess IL-EG in the RPUF matrix results in agglomeration. Therefore, the horizontal burning rate of the P4 and P5 composites increased, compared to that of RPUF.

### 3.6. Char Residue Analysis and Flame-Retardant Mechanism

**Figure 9:**
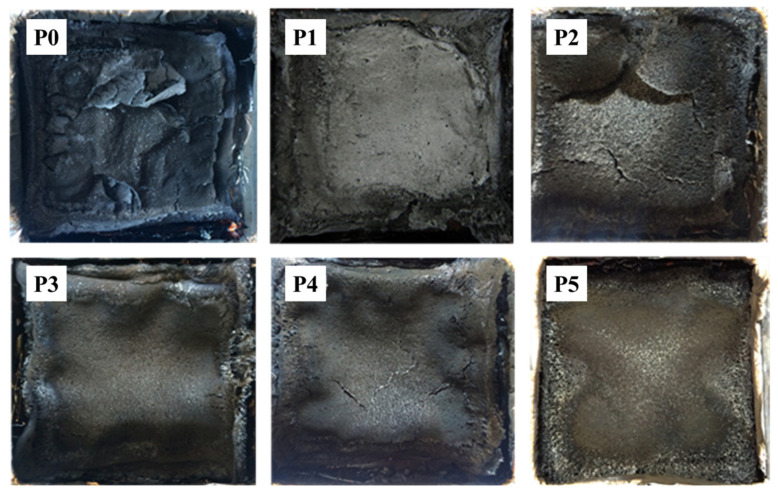
Digital photos of carbon residue after burning of the RPUF samples.

Figure 9 shows the digital images of the char residue after the CONE test. From the figure shown, it is apparent that a thin residual char layer formed after the pure RPUF burned, and the char layer surface was severely cracked, providing limited resistance to heat or gas and resulting in poor flame-retardance. The addition of DPES significantly reduced the cracks on the surface of the residual char layer that formed after burning the RPUF, resulting in a thicker residual char layer with enhanced surface integrity. For RPUFs containing both IL-EG and DPES, the morphology of the surface of the residual char layer of P2, P3 and P4 showed that even in the presence of cracks, the residual char layer became clear, and the surface densification, the layer thickness and the resistance of the residual char layer to heat or smoke were all increased. After combustion of a large quantity of EG on the PU surface, the formation of an intact and stable residual char layer can inhibit the exchange of heat or gas from the matrix interior to the exterior, thus improving the flame retardancy of RPUF. When the amount of IL-EG was in excess or insufficient, cracks were clearly observed on the surface of the residual char layer, which decreased the compactness and strength of the residual char layer. When RPUF containing only IL-EG was burned, the residual char layer of P5 effectively maintained the density and integrity of the surface at a certain layer thickness, but the char layer surface here was less smooth than that of P3, indicating that the combination flame-retardant system was more conducive to the formation of the residual char layer. This result was attributed to the action of an appropriate amount of DPES on RPUF, whereby a *π–π* bond facilitated internal interwoven hydrogen bonding among the IL, EG and DPES molecules, resulting in the successful formation of a new flame-retardant covering on the PUF surface. When PUF burns, the graphite in the outer layer of the foam expands to form an insulating graphite layer, which has a synergistic effect with the noncombustible gas produced by the combustion of the phosphorus contained in the IL. The Si-C bond that forms simultaneously between the Si and C in DPES results in a char layer with a high strength and good barrier properties after burning. In this case, the optimum effect of the combination flame-retardant is obtained, significantly enhancing the flame retardancy of the RPUF.

**Figure 10:**
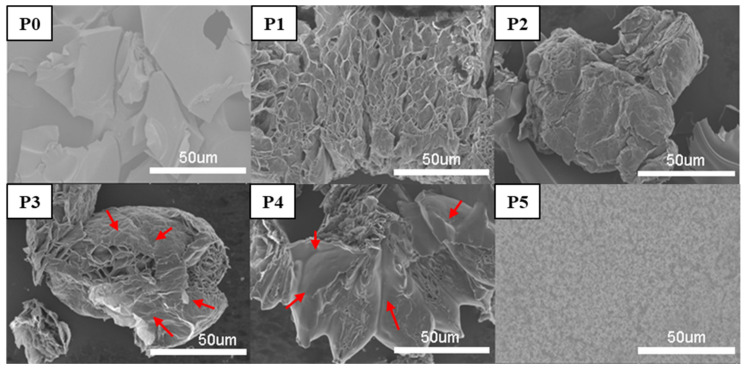
SEM photos of the residue char after the cone calorimeter test.

SEM images of the char residue of RPUF and flame-retardant RPUFs after burning are shown in Figure 10. The use of IL-EG as a flame-retardant alone had a limited expansion effect, whereas the corresponding use of DPES resulted in an expanded char layer with a porous network structure that facilitated the diffusion of heat and gas. The combination IL-EG/DPES flame-retardant used with RPUFs resulted in a char residue that facilitated an expanded network structure, while covering the surface with a smooth film of a closed char layer (as the red arrows point to in P3 and P4) that effectively blocked the transmission of gas and heat, and improved RPUF flame-retardancy [35].

## 4. Conclusions

The synergistic effect of a combination flame-retardant IL-EG/DPES on the structure and flame retardancy of RPUF was investigated in this paper. The results showed that a 1:1 ratio of DPES and IL-EG maximized the compressive strength and optimized the flame retardancy of RPUF over that of a RPUF modified with only DPES or IL-EG. The PHRR, THR, smoke release rate (SRR) and TSR of the flame-retardant RPUF were correspondingly significantly decreased, and the LOI and TTI of the synergistic flame-retardant were correspondingly significantly increased. A closed char layer forms on the surface of the RPUF modified with the synergistic flame-retardant, which effectively blocks the transmission of gas and heat and improves RPUF’s flame-retardancy.

## Figures and Tables

**Table 1:** Formulations of RPUF/IL-EG composites.

Sample	P0 (phpp ^a^)	P1 (phpp)	P2 (phpp)	P3 (phpp)	P4 (phpp)	P5 (phpp)
HF-4110H	70	70	70	70	70	70
HF-4110	30	30	30	30	30	30
H_2_O	3	3	3	3	3	3
AK-8803	2	2	2	2	2	2
A33	2	2	2	2	2	2
GI	1.0	1.0	1.0	1.0	1.0	1.0
PAPI	138	138	138	138	138	138
IL-EG	0	0	5	10	15	20
DPES	0	20	15	10	5	0

**^a^** per hundred of polyether polyol by weight.

**Table 2:** Theoretical and measured values of the content of each element in DPES.

Element	C	H	N	P	Si
Theoretical value (wt %)	61.37	7.97	4.03	7.67	6.93
Measured value (wt %)	61.03	7.72	3.64	7.50	7.05

**Table 3:** TG and DTG data for the various samples.

Sample	T_5%_ (°C)	T_10%_ (°C)	T_50%_ (°C)	T_onset_ (°C)	T_max_ (°C)	Residue at 700 °C (wt%)
P0	196.5	249.8	364.8	180.0	356.5	17.3
P1	145.9	223.4	348.4	125.7	315.1	17.9
P2	210.7	256.7	345.7	196.7	321.2	22.2
P3	205.2	255.2	340.2	188.4	320.7	24.1
P4	203.6	242.3	333.5	189.4	314.4	31.9
P5	171.1	259.8	357.3	150.0	352.0	19.3

**Table 4:** Flame-retardant RPUF cone calorimetric analysis data.

Sample	TTI (s)	PHRR (kW/m^2^)	THR (MJ/m^2^)	TSR (m^2^)
P0	4 ± 1	243 ± 15	21.8 ± 5	11.6 ± 2
P1	7 ± 2	190 ± 11	16.8 ± 3	8.7 ± 1
P2	8 ± 2	143 ± 10	14.6 ± 4	6.7 ± 1
P3	10 ± 1	96 ± 6	12.0 ± 1	3.6 ± 0.5
P4	9 ± 2	111 ± 9	13.2 ± 3	4.9 ± 1
P5	8 ± 2	108 ± 7	13.9 ± 3	4.1 ± 1

**Table 5:** Horizontal combustion data of flame-retardant RPUF composites.

Sample	P0	P1	P2	P3	P4	P5
Horizontal burning rate/mm·min^−1^	51.5 ± 9	55.9 ± 8	41.7 ± 6	25.6 ± 3	33.6 ± 4	37.2 ± 3

## Supplementary Materials

The following are available online at https://www.mdpi.com/1996-1944/13/14/3095/s1, Figure S1: ^1^H-NMR spectra of (**a**) bromide-1-butyl-3-methyl imidazole, (**b**) hydroxide-1-butyl-3-methyl imidazole and (**c**) butyl-3-methyl-imidazole phosphate. (0.871(1H); 1.204 (2H); 1.723 (3H); 4.094 (4H); 8.633 (5H); 7.349 (6H); 7.398 (7H); 3.812 (8H)). Figure S2: DTG curves of RPUF modified with different content of IL-EG/DPES. Figure S3: FTIR analysis of gas escape for RPUF and IL-EG/DPES flame-retardant RPUF at the point of maximum weight loss rate: (**a**) P0, (**b**) P1, (**c**) P2, (**d**) P3, (**e**) P4 and (**f**) P5.
