# Supplementary material for: The Synergistic Effect of Ionic Liquid-Modified Expandable Graphite and Intumescent Flame-Retardant on Flame-Retardant Rigid Polyurethane Foams
Source: Materials (Basel). 2020 Jul 10;13(14):3095. doi: 10.3390/ma13143095 (PMC7411720; doi:10.3390/ma13143095)
Supplement: Supplementary file 1 [file materials-13-03095-s001.pdf]

materials

MDPI

Article

The Synergistic Effect of Ionic Liquid-Modified Expandable Graphite and Intumescent Flame-Retardant on Flame-Retardant Rigid Polyurethane Foams

Yongjun Chen [1,*], Yuanfang Luo [1], Xiaohui Guo [1], Lijuan Chen [2,*] and Demin Jia [1]

1. College of Materials Science and Engineering, Key Lab of Guangdong Province for High Property and Functional Macromolecular Materials, South China University of Technology, Guangzhou 510641, China; psyfluo@scut.edu.cn (Y.L.); msgxh@mail.scut.edu.cn (X.G.); psdmjia@scut.edu.cn (D.J.)
2. Center for Advanced Analytical Science, c/o School of Chemistry and Chemical Engineering, Guangzhou University, Guangzhou 510006, China

* Correspondence: yjchen@scut.edu.cn (Y.C.); gdchenlj@gzhu.edu.cn (L.C.); Tel.:+86-020-87114857

Received: 19 May 2020; Accepted: 7 July 2020; Published: date

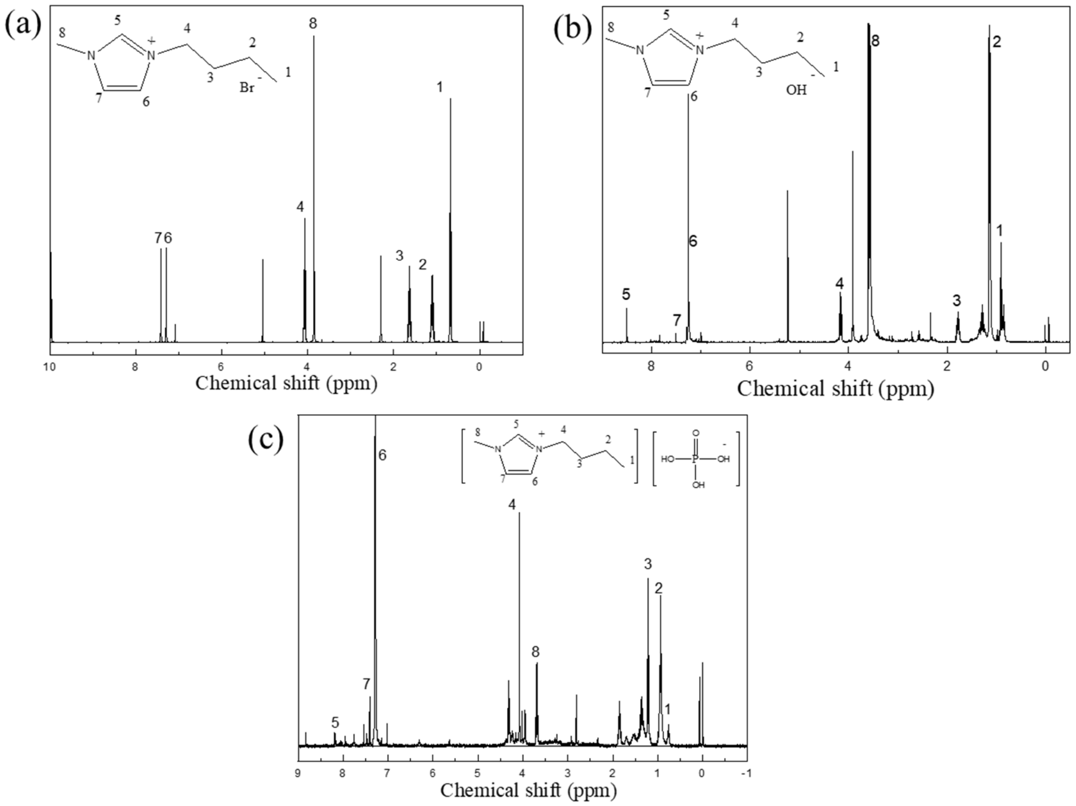

Figure S1. 1H-NMR spectra of (**a**) bromide-1-butyl-3-methyl imidazole, (**b**) hydroxide-1-butyl-3-methyl imidazole and (**c**) butyl-3-methyl-imidazole phosphate. (0.871(1H); 1.204 (2H); 1.723 (3H); 4.094 (4H); 8.633 (5H); 7.349 (6H); 7.398 (7H); 3.812 (8H)).

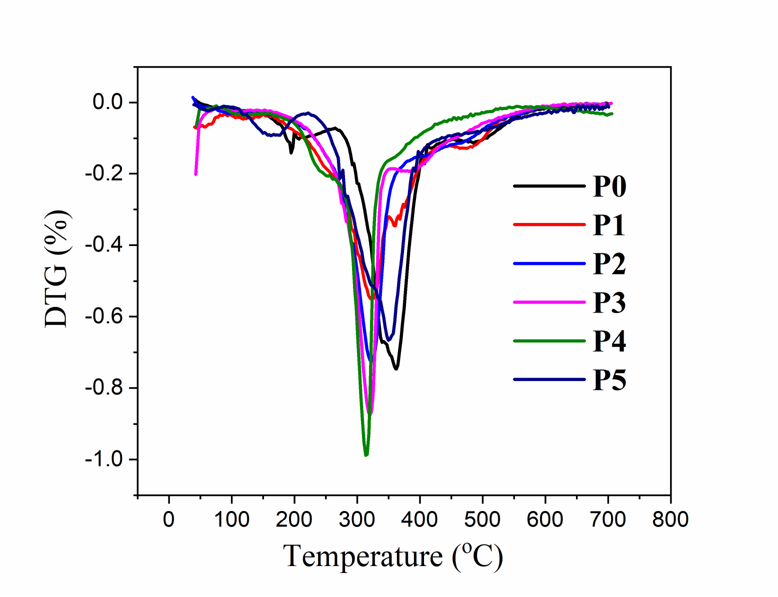

Figure S2. DTG curves of RPUF modified with different content of IL-EG/DPES.

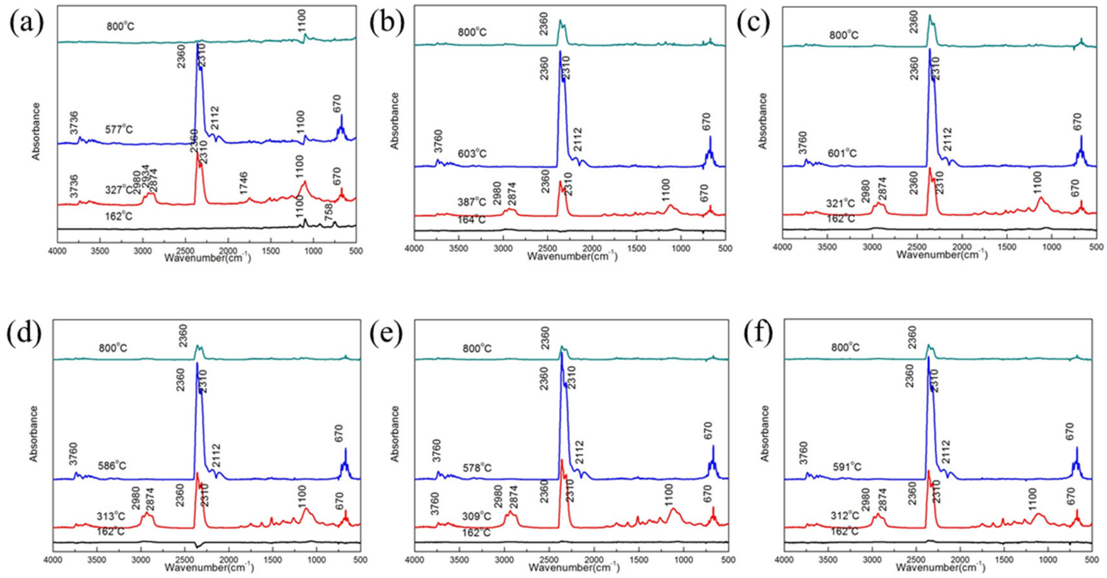

Figure S3. FTIR analysis of gas escape for RPUF and IL-EG/DPES flame-retardant RPUF at the point of maximum weight loss rate: (**a**) P0, (**b**) P1, (**c**) P2, (**d**) P3, (**e**) P4 and (**f**) P5.

© 2020 by the authors. Submitted for possible open access publication under the terms and conditions of the Creative Commons Attribution (CC BY) license (http://creativecommons.org/licenses/by/4.0/).
